# Unlocking High‐Voltage Aqueous LiNi_0.5_Mn_1.5_O_4_ Cathodes Through Binder‐Collector Interfacial Design

**DOI:** 10.1002/smsc.70406

**Published:** 2026-09-25

**Authors:** Giorgio Montinaro, Daniele Versaci, Piera Di Prima, Julia Amici, Federico Bella, Silvia Bodoardo

**Affiliations:** ^1^ Department of Applied Science and Technology Politecnico di Torino Turin Italy

**Keywords:** aqueous processing, binder, cathodes, LiNi_0.5_Mn_1.5_o_4_, lithium‐ion battery

## Abstract

Lithium‐ion battery performance and sustainability increasingly depend on “inactive” components such as polymer binders and current collectors. Replacing conventional N‐methyl‐2‐pyrrolidone/polyvinylidene fluoride processing with water‐based manufacturing requires binders that combine mechanical cohesion, interfacial stability, and ionic transport, together with current collectors resistant to corrosion and high‐voltage degradation. Here, two aqueous binder formulations—sodium carboxymethyl cellulose (NaCMC)  +  SX8684(A)‐64 and NaCMC  +  TRD202A—are investigated in LiNi_0__.__5_Mn_1__.__5_O_4_ (LNMO) cathodes cast on bare and carbon‐coated aluminum foils. Spectroscopic and thermogravimetric analyses identify SX8684(A)‐64 as a single‐phase polymethacrylate with minimal swelling (~2%–3% mass uptake), whereas TRD202A exhibits a hybrid acrylic/fluoropolymer architecture, multistage degradation, and pronounced gel‐like swelling (>30%). Morphological analysis shows porosities of 67%–71% and tortuosities of 2.4–3.7, with TRD202A on carbon‐coated aluminum producing the most open and least tortuous architecture. Intermittent current interruption measurements confirm that this configuration provides the lowest resistance, while delivering >100 mAh g^−1^ at 5 C, >45  mAh  g^−1^ at 15 C, and >80% capacity retention over 100 cycles. These results demonstrate the importance of co‐designing binder chemistry and current‐collector surfaces for scalable, sustainable aqueous LNMO cathodes.

## Graphical abstract

Aqueous processing of high‐voltage LNMO cathodes is enabled by co‐designing binder chemistry and current collector interfaces. The study shows that a hybrid acrylic/fluoropolymer binder combined with carbon‐coated aluminum forms crack‐free, low‐resistance electrodes with improved ion transport, high‐rate capability up to 15 C, and stable cycling, offering a greener route to cobalt‐free lithium‐ion batteries.

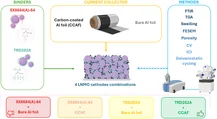

## 1 Introduction

Electrochemical energy‐storage systems, particularly lithium‐ion batteries (LiBs), have become the technology of choice for portable electronics, stationary grid‐scale storage, and electric vehicles due to their superior energy and power densities [1, 2]. However, with demands for faster charging, longer runtimes, and wider application scopes continually rising, both energy and power performance improvements remain essential [3, 4]. Among the active materials driving these advances, lithium iron phosphate (LiFePO_4_) has been widely adopted for its outstanding thermal stability and long cycle life [5, 6, 7], nickel‐rich layered oxides such as LiNi*_x_* Mn_*y*_Co_1−*x*−_ *_y_* O_2_ (NMC) offer high energy density and tunable electrochemical properties via compositional adjustments [8, 9], and lithium nickel cobalt aluminum oxide (NCA) provides high specific capacity at a moderate cost, making it a mainstay in automotive powertrains [10]. More recently, high‐voltage spinel LiNi_0__.__5_Mn_1__.__5_O_4_ (LNMO) has emerged as a cobalt‐free alternative with a ~4.75 V operating plateau and a theoretical capacity of 146.7 mAh g^−1^, promising lower cost and environmental footprint [11]. While these advances in active materials and cell‐manufacturing techniques—optimizing slurry formulations, coating methods, and calendaring conditions—have enhanced electrode uniformity and ionic transport pathways [12], it is now broadly acknowledged that the so‐called “inactive” components, such as polymeric binders, conductive additives, and current collectors, account for up to 60% of cell mass and critically determine rate capability, energy density, and long‐term stability [13, 14]. For instance, binders govern particle cohesion, film‐forming behavior, and interfacial stability [13], while current collectors must deliver robust electronic contact and resist corrosion [7, 12]. In this regard, surface modification and engineering in current collectors play a pivotal role as well: bare aluminum foils are prone to corrosion and interfacial delamination under high‐voltage and aqueous processing conditions, whereas carbon‐coated aluminum foils form a protective conductive interlayer that enhances adhesion, suppresses corrosion, and reduces interfacial resistance by up to 50% [15]. In the context of waterborne cathode processing, coupling CMC‐based binders with carbon‐coated collectors enables the use of more environmentally friendly aqueous slurries without sacrificing electrochemical stability, opening the door for sustainable high‐voltage LNMO cell fabrication.

Electrode‐slurry formulation exerts a profound influence on both cell performance and manufacturing sustainability, driving recent research toward alternative binder chemistries that must balance mechanical cohesion, interfacial adhesion, electrochemical and thermal stability, minimal swelling in electrolyte, and unobstructed ion transport—while evenly contributing to electronic conductivity and both solid/electrolyte and cathode/electrolyte interphases (SEI and CEI) layers stabilization [13, 16]. Polyvinylidene fluoride (PVdF) has long dominated as the binder of choice due to its chemical robustness and processability [17, 18, 19], but its reliance on toxic vinylidene fluoride monomers and N‐methyl‐2‐pyrrolidone (NMP) solvent raises serious health, environmental, and regulatory concerns, also due to the energy‐intensive recovery process requirements. Moreover, conventional PVdF/NMP‐based slurry preparation is roughly 26 times more expensive than aqueous methods, with NMP priced at 1–3 USD kg^−1^ and PVdF at 8–10 USD kg^−1^ versus water at <0.02 USD L^−1^ and polysaccharide binders like carboxymethyl cellulose (CMC) at 2–5 USD kg^−1^ [20]. Transitioning to water‐based binders not only slashes raw‐material costs, but also reduces energy consumption by over 40%—by eliminating the solvent‐recovery step—and aligns with green‐manufacturing principles under REACH regulations that increasingly restrict NMP use [13, 20]. As a result, water‐soluble polysaccharides (e.g., CMC and sodium alginate) and emerging bio‐based or conductive polymeric binders have garnered growing interest in delivering comparable or superior electrode integrity, interfacial stability, and cost‐environmental benefits [21].

Interest in aqueous polysaccharide binders has also surged owing to their ability to form uniform films in water‐based slurries, enhancing both electrode integrity and sustainability. CMC, a linear cellulose derivative rich in hydroxyl and carboxyl groups, promotes stable SEI/CEI layer formation and creates cross‐linked networks between active particles [22, 23]. When its sodium salt (NaCMC) is paired with styrene‐butadiene rubber (SBR), the resulting NaCMC/SBR system delivers superior dispersion, flexibility, binding strength, and processability, making it the benchmark aqueous binder for—as an example— graphite anodes [19]. Beyond CMC, other natural polymers have demonstrated promising binder roles: sodium alginate offers robust film formation and ion–dipole interactions for improved cohesion [24], arabic gum (GA), composed of polysaccharides and glycoproteins, provides mechanical tolerance against volume changes [25], xanthan gum (XG) with its double‐helix structure enhances electrode integrity [26], guar gum ensures uniform coverage and mitigates capacity fade [27], and carrageenan has been shown to function as “sacrificial” binders in high‐‍voltage spinels, uniformly covering LNMO particles and stabilizing the cathode–electrolyte interphase [28]. Chitosan‐based binders, engineered for hyper‐branched flexibility and adhesion, further expand the aqueous binder toolkit [29]. Synthetic water‐soluble polymers have also been explored: polyacrylic acid (PAA) improves ionic transport and reduces resistance [30], acrylic‐latex copolymers deliver thermal and electrochemical stability [31], poly(vinyl alcohol)/PAA blends balance adhesion and porosity [32], and high‐molecular‐weight polyacrylonitrile (PAN) variants show promise for water‐based electrode processes [33]. In parallel, composite binder strategies (e.g., PAA‐SA) have demonstrated capacity retentions exceeding 90% after 100 cycles at 0.5 C, underscoring the promise of tailored aqueous formulations [34]. Despite these advances, making aqueous binders the state of the art for anode fabrication, adopting water‐based processes for high‐voltage cathodes—such as LNMO—remains challenging, and fluorinated polymers like PVdF still predominate in cathode manufacturing.

Although LNMO appeals as a cobalt‐free, high‐voltage spinel cathode is well established, its ~4.7 V operating plateau accelerates the oxidative breakdown of conventional polymer binders and aggravates side reactions—such as lithium leaching and aluminum foil corrosion—thereby making water‐based electrode processing particularly challenging [35, 36]. Early demonstrations revealed that water‐soluble CMC can yield higher initial capacities, but suffers inferior long‐term cyclability versus PVdF/NMP systems [34]. Since then, a broad spectrum of aqueous binders has been explored, as previously stated [37, 38].

Beyond the specific chemistries investigated herein, a broader conceptual shift is required in high‐voltage cathode design: binders and current collectors should no longer be treated as passive auxiliaries, but rather as functional, co‐engineered components that actively dictate interfacial kinetics, mechanical resilience, and ionic percolation. In 5  V‐class spinels such as LNMO, where oxidative stress, electrolyte instability, and metal dissolution are intrinsically amplified, the electrode architecture must be conceived as an integrated system in which polymer chemistry, collector surface energy, and pore‐network topology are synergistically optimized. Establishing such a rational co‐design framework is essential not only for performance enhancement, but also for enabling scalable, water‐based manufacturing routes that remain competitive with conventional PVdF/NMP processing.

In this study, we systematically evaluate two aqueous binder formulations—NaCMC  +  TRD202A and NaCMC  +  SX8684(A)‐64—applied to high‐voltage LiNi_0__.__5_Mn_1__.__5_O_4_ cathodes to elucidate a possible optimal binder chemistry for electrode integrity and electrochemical performance. Each binder system is tested on both bare aluminum and carbon‐coated aluminum (CCAF) current collectors to assess the synergistic impacts of binder‐collector interface engineering on resistance, rate capability, and cycling stability. NaCMC, widely recognized for its film‐forming ability and SEI/CEI‐stabilizing functional groups, provides excellent particle cohesion and electrolyte compatibility in aqueous slurries, while TRD202A—a fluorinated‐acrylic hybrid latex—has demonstrated enhanced coating uniformity and cycle retention in various high‐voltage cathodes [39], and SX8684(A)‐64, a styrene‐butadiene‐based polymethacrylate emulsion, imparts superior mechanical flexibility and adhesion under fast‐charge conditions [40]. Notably, TRD202A has previously demonstrated favorable long‐term stability when applied to Li‐rich layered oxide cathodes, outperforming conventional PVdF‐based films after extended cycling [41]. CCAF collectors have been shown to mitigate foil corrosion in water‐based processing, reducing interfacial impedance by 20%–50%, and enabling stable LNMO performance even at >1 C rates [33, 42]. By comparing rate‐capability profiles, intermittent current interruption (ICI) trends, and microstructural analyses across these four configurations—CMC + TRD202A on bare and coated foil, and CMC + SX8684(A)‐64 on bare and coated foil—in the present study, we aim to identify the optimal binder‐collector combination among these four for sustainable, high‐performance aqueous LNMO cathode manufacturing.

## 2 Experimental Section

### 2.1 Chemical and Electrochemical Characterization of TRD202A and SX8684(A)‐64

TRD202A and SX8684(A)‐64 (JSR Corporation, Japan) were used as received for standalone characterization. Both are classified as water‐based acrylic emulsions, although their precise chemical structure and formulation are not disclosed. The two binders exhibit comparable physical properties, with a (solution) density of 1.01 g cm^−3^ and a solid content of 41.3 wt%.

Chemical characterization of the two specimens was carried out by Fourier‐transform infrared/Attenuated total reflection (FTIR/ATR) spectroscopy, which was performed by means of a Nicolet iS50 FTIR spectrometer (Thermo Fisher Scientific Inc., Waltham, MA, United States), equipped with a built‐in mid‐IR diamond ATR module ranging from 4000 to 500 cm^−1^ wavelength. Samples were prepared by pouring the two liquid binders into circular silicone molds (∅  ~  5 cm), and then dried in an oven (UF55, Memmert, Germany) at 50 °C for 2 h. Afterward, they were left to dry further under a ventilated hood overnight. Once fully dried, self‐standing binder disks with an area of 0.785 cm^2^ (∅ 10 mm) were punched out from the molds and vacuum‐dried using a Büchi Glass Oven B‐585 (Switzerland) at 50 °C for 48 h before proceeding to the FTIR/ATR analyses.

An analogous procedure was used to prepare the binder samples for the swelling test. Self‐standing disks (∅ ~2.5 cm) were punched from the same circular silicon molds described above, and their initial thickness and masses were recorded. After drying overnight under a ventilated hood and then under vacuum at 50 °C for 48 h, the disks were transferred into the glovebox. In labeled petri dishes, each disk was immersed in 1 mL of LP40 electrolyte (1 M LiPF_6_ in EC:DEC 1:1 v/v; Solvionic, Toulouse, France) for 24 h. Finally, the thickness and mass of each disk were remeasured to determine volumetric expansion and electrolyte uptake, from which the swelling ratio was calculated.

Thermogravimetric analysis (TGA) was carried out on a TG 209 F3 Tarsus (NETZSCH‐Gerätebau GmbH, Selb, Germany) to evaluate binder stability and degradation pathways. Approximately 7–10 mg of binder was loaded into alumina crucibles, and each formulation was analyzed in duplicate. For the inert measurements, samples were heated from 25 to 800 °C at 10 °C min^−1^ under a nitrogen purge (60 mL min^−1^) to isolate thermal decomposition in the absence of oxygen in order to simulate a real cell environment. The same heating program (25–800 °C at 10 °C ‍min^−1^) was applied under an air flow (60 mL min^−1^) to reveal additional degradation events—such as oxidative cross‐linking and combustion of char residues. Baseline correction and derivative weight‐loss curves were used to pinpoint onset temperatures and decomposition stages for each binder.

Electrochemical characterization—in terms of oxidative and general electrochemical stability—of the two specimens was carried out by cyclic voltammetry (CV) using a VMP‐3e versatile multichannel potentiostat (Biologic, Grenoble, France). Both TRD202A and SX8684(A)‐64 samples were prepared by tape casting the liquid binders on carbon‐coated aluminum foil (MTI Corporation, CA, United States, battery grade) using an automated film applicator (AB4120, TQC Sheen, Netherlands, speed: 20 mm s^−1^) and a micrometer adjustable doctor blade (SeKTQ125, MTI Corporation, thickness: 100 μm). The obtained sheets were first dried in an oven at 50 °C for 2 h. Circular electrodes (∅ 10 mm, 0.785 cm^2^) were punched and subjected to an additional drying step in a vacuum oven (80 °C, 4 h). The dried disks were then transferred into a glovebox (MBraun Labstar, Germany; H_2_O < 0.5 ppm and O_2_ < 50 ppm) for the assembly of conventional three‐electrode Swagelok t‐cells used for CV analysis. Each cell included glass fiber separators (Whatman GF/D, Germany, ∅ 10 mm), soaked with 200 μL of LP40 electrolyte, and two lithium disks (∅ 10, 0.3 mm‐thick, high‐purity lithium foils, Chemetall Foote Corp., United States), used as counter and reference electrodes. CV testing was conducted in a 3–5 V potential range, at ambient temperature, and with a fixed scan rate of 0.1 mV s^−1^, for 5 cycles (bandwidth: 5).

### 2.2 Electrode Manufacturing

For both binder types (TRD202A and SX8684(A)‐64) and for both current collector configurations (bare aluminum foil and CCAF) used in this study, the cathodic slurry formulation was kept identical in terms of weight ratio: 92:3:1:4, corresponding to active material, conductive additive, NaCMC, and either TRD202A or SX8684(A)‐64, respectively. This ratio was selected on the basis of preliminary formulation screening and processability considerations (slurry viscosity and film‐forming behavior) and was kept fixed across all formulations investigated in this study. The selected active material was LiNi_0__.__5_Mn_1__.__5_O_4_ NANOMYTE SP‐10 (NEI Corporation, Somerset, NJ, USA), used as received. The conductive additive employed was C‐NERGY SUPER C45 carbon black (Imerys, Paris, France), which was also used as received. NaCMC (CMC 1220, average *M*_w_: 2.3 × 10^5^ Da) was supplied by Daicel Miraizu Ltd. (Nakashibetsu, Japan). For the specifics on TRD202A and SX8684(A)‐64, please refer to the previous section.

For slurry preparation and mixing, an overhead stirrer with adjustable speed (up to 2200 rpm) was used (AM 20‐D ARGOlab Overhead Stirrer, ARGOlab, Modena, Italy). First, CMC 1220 was dissolved in Milli‐Q water (Arium Mini water purification system, Sartorius AG, Göttingen, Germany) and stirred for up to 30 min to ensure complete dissolution. The conductive additive was then added and mixed for an additional 30 min. Once fully dispersed, the active material was introduced, and the mixture was stirred for 4 h. Finally, either TRD202A or SX8684(A)‐64 was added and stirred at reduced speed to avoid mechanical degradation for approximately 1 h. The resulting slurry exhibited a solid content of approximately 52% by weight. Each prepared slurry was then cast onto either carbon‐coated aluminum foil or bare battery‐grade aluminum foil (AL‐1085, Hitachi, Japan, >99% purity) using an automated film applicator (speed: 10 mm s^−1^) and a micrometer‐adjustable doctor blade set to a thickness of 300 μm. The resulting cathode sheets were dried in an oven at 50 °C for 1 h. After drying, electrodes were punched into disks of 1.76 and 0.77 cm^2^ (∅ 15 and 10 mm, respectively), intended for use in coin cells and Swagelok t‐cells. The punched electrodes underwent a final drying step in a vacuum oven (120 °C, 4 h, 10 mbar) before cell assembly in a glovebox environment. The final active material mass loading of the prepared electrodes ranged between 10 and 12 mg cm^−2^.

### 2.3 Electrochemical and Morphological Characterization of the Electrodes

CV on the fabricated electrodes was performed following a protocol analogous to that used for the binder coatings. Electrode disks (10 mm Ø, 0.785 cm^2^) were punched out and subjected to an additional drying step in a vacuum oven at 80 °C for 4 h. Conventional three‐electrode Swagelok T‐cells were assembled using the same protocol and components used during binder testing. The separators were wetted with 200 µL of the LP40 electrolyte. CV scans were then run between 3 and 5 V at room temperature using a scan rate of 0.1 mV s^−1^ for five consecutive cycles. The cycling performance (rate capability and capacity retention) was evaluated in a half‐cell configuration by assembling 2032‐type coin cells. Lithium metal disks (Ø 16, 0.6 mm thick, Tobmachine, China) were used as counter electrodes. Glass fiber separators (Ø 18 mm) were soaked with 150 μL of LP40 electrolyte solution. All galvanostatic charge–discharge tests were performed at room temperature using an Arbin LBT21084 battery tester (Arbin Instruments, TX, USA). The voltage window was set to 3.0–4.9 V versus Li^+^/Li, and charge/discharge rates were calculated based on the nominal specific capacity of LNMO at C/10, as indicated by the product specification sheet provided by the producer (i.e., 125 mAh g^−1^). The cycling protocol consisted of three initial cycles at C/10 (charge and discharge), followed by five cycles at C/5, and five cycles at 1 C. Subsequently, rate capability was assessed through a sequence of three five‐cycle loops in which the charge rate was fixed at 1 C, while the discharge rate progressively varied (5, 10, and 15 C). Finally, three cycles at C/10 preceded a long‐term cycling test of 100 cycles at 1 C (symmetrical charge and discharge).

ICI testing, performed as an alternative to potentiostatic electrochemical impedance spectroscopy (PEIS) and to galvanostatic intermittent titration technique (GITT) [43, 44, 45], was conducted in parallel with galvanostatic cycling to evaluate differences in both apparent diffusion coefficients and—specifically—internal resistances among the systems under investigation. Cell assembly followed the same procedure described for galvanostatic cycling using identical components and electrolyte volumes. Following a five‐cycle formation step at C/10 (symmetrical charge and discharge) within the 3.0–4.9 V potential window, the ICI protocol was applied using 5 min current pulses at C/10, each followed by 5 s rest intervals. During these rest periods, the cell potential was recorded at 0.1 s intervals. The resulting potential profiles were analyzed through fitting and modeling using MATLAB R2024a (MathWorks, MA, United States), as described in references [44, 45].

Morphological characterization of the samples was performed using field emission scanning electron microscopy (FESEM, Zeiss SUPRA 40, Italy), equipped with a Gemini column and a Schottky field‐emission tip (tungsten at 1800 K). All four electrode configurations—combining the two water‐based acrylic binders with the two aluminum current collectors—were analyzed through both surface and cross‐sectional FESEM imaging. Micrographs were acquired at various magnifications, ranging from 500 x to 10 kx. For porosity and tortuosity estimation, five images were taken at different regions of each sample in cross‐section at a fixed magnification of 10 kx. Image analysis was conducted using MATLAB R2024a, following the procedure described in [46, 47]. A mean value of porosity per sample class was obtained by averaging the porosity value obtained by each of the five images.

## 3 Results and Discussion

FTIR/ATR analyses were performed on both SX8684(A)‐64 and TRD202A samples to reveal their chemical compositions and identify the key functional groups present in each binder. The corresponding spectra are shown in Figure 1. Based on previous literature and manufacturer technical sheets, the chemical structures of these binders have been proposed, although their precise molecular compositions remain unconfirmed. SX8684(A)‐64 is likely a water‐based styrene‐butadiene polymethacrylate emulsion [40], while TRD202A is supposed to be a hybrid polymer water‐based emulsion blending an acrylic polymer with a fluoropolymer component [48].

**FIGURE 1:**
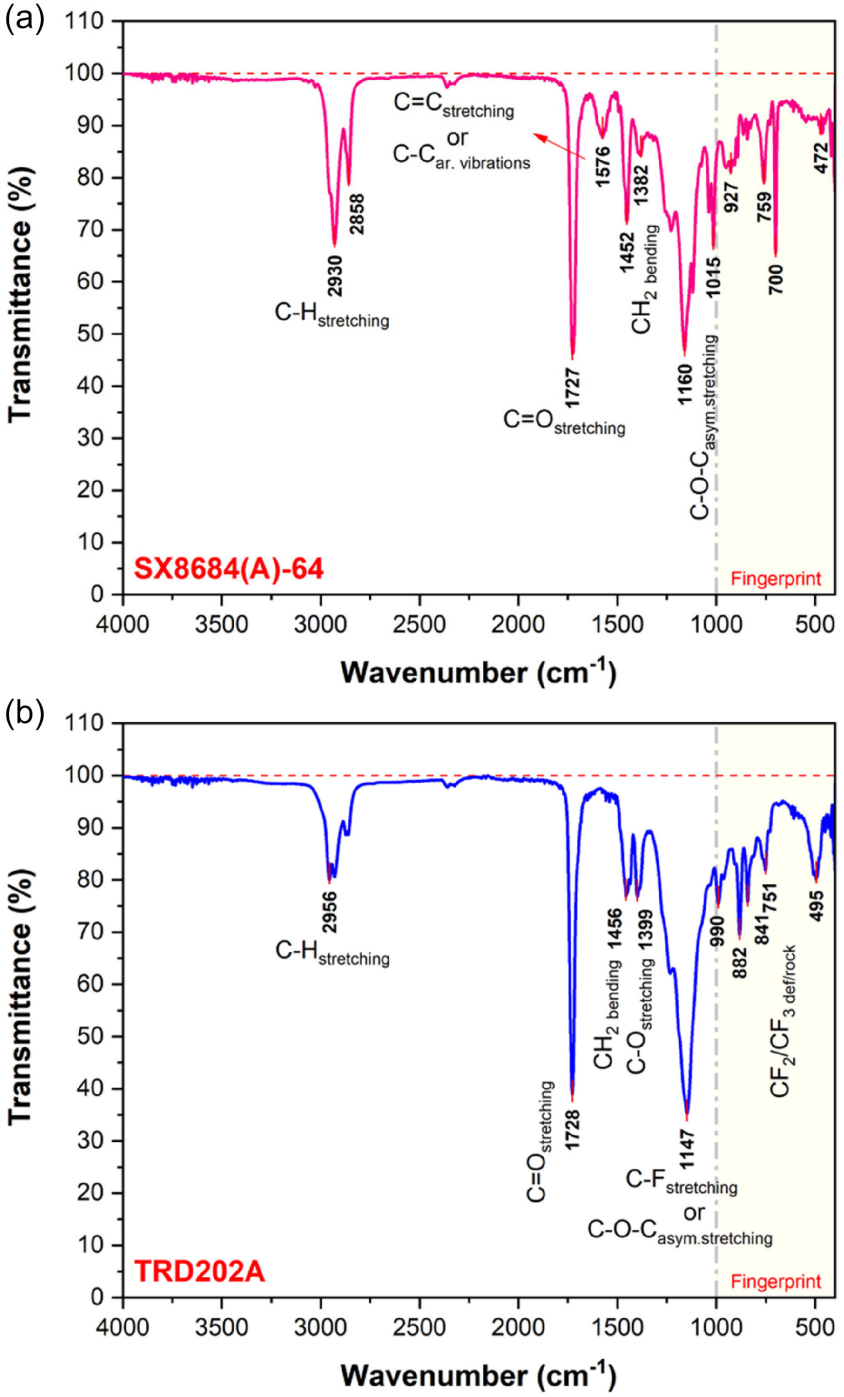
FTIR‐ATR spectra of LNMO cathode binders: (a) SX8684(A)‐64 and (b) TRD202A.

Both spectra exhibit strong absorptions around 1727–1728 and 1147–1160 cm^−1^ corresponding to carbonyl (C═O) stretching and ether (C─O─C) asymmetrical stretching from ester functionalities‍—a signature commonly associated with acrylic or methacrylic polymers [49]. This supports the presence of polyacrylate or polymethacrylate backbones in both binders.

The spectrum of SX8684(A)‐64 (Figure 1a) presents more absorptions in the 927–1159 cm^−1^ range, associated with O─CH_3_ rocking and C─O─C symmetric stretching, suggesting the presence of side chains rich in ester or ether linkages. These features, together with moderate bands at ~1381 and ~1452 cm^−1^ (CH_3_ and CH_2_ bending modes), are characteristic of polymethacrylates such as poly(methyl methacrylate) (PMMA) or its copolymers [50]. The spectral profile is consistent with a binder rich in polar functional groups, which could enhance hydrophilicity and adhesion to active material surfaces, but may also interact differently with the aqueous processing environment or high‐voltage cathode chemistries [51]. Low‐intensity absorptions at ~759 and ~700 cm^−1^ can coincide both with C─C skeletal vibrations or with out‐of‐plane aromatic C─H bending modes characteristic of styrene‐like units, suggesting minor styrene incorporation or processing impurities. The presence of aromatic moieties, however, cannot be confirmed due to the absence of characteristic peaks in the spectra (e.g., aromatic stretch near 3100–3000 cm^−1^ or strong intensity C═C in‐plane ring vibrations 1600–1400 cm^−1^), thus needing further characterization to be ascertained. Taken together, these assignments support describing SX8684(A)‐64 as a waterborne styrene‐polymethacrylate emulsion—rich in polar ester groups for adhesion—rather than a pure SBR system. Finally, the lack of strong intensity SBR‐specific absorptions at ~966 cm^−1^ (trans‐C─H wag) and ~911 cm^−1^ (cis/mono‐substituted C─H wag) further supports this conclusion [52].

By contrast, the spectrum of TRD202A (Figure 1b) shows slightly stronger C─O bands and a slightly weaker aliphatic (methyl, CH_3_) stretching feature around 2956 cm^−1^, which may suggest the influence of different functional groups in comparison to what was observed in the case of SX8684(A)‐64 and a more hydrophobic polymeric environment [16]. These features are hypothetically consistent with a hybrid formulation combining an acrylic polymer with a fluoropolymer fraction as fluoropolymers typically reduce the density of polar vibrational modes while also reducing the proportion of hydrocarbon‐like features (e.g., C─H stretching at high wavenumbers). However, the absence of distinct C─F stretching vibrations, typically expected in the 1100–1300 cm^−1^ region, implies that any fluorinated segments are either present in low concentration or—more likely—structurally integrated in a way that masks their spectral signature [53]. The broader and less intense spectral features (in particular, the C─O─C asymmetrical stretching band in which the C─F stretching could be confounded) may also reflect increased heterogeneity or phase separation within the polymer matrix, a behavior not uncommon in hybrid systems comprising acrylic and fluorinated components.

These observations sustain the hypothesis that SX8684(A)‐64 behaves as a conventional polymethacrylate emulsion, whereas TRD202A functions as a hybrid binder combining an acrylic backbone with fluoropolymer‐derived domains. Such differences in chemical structure are not merely compositional—they can have important consequences for the interfacial chemistry between the binder and the LNMO active material.

Specifically, the functional groups identified by FTIR, such as the carbonyl (C═O) stretches and C─O/C─O─C vibrations, are of paramount importance in dictating how binders adhere to and interact with the LNMO particle surface. In a conventional polymethacrylate system like SX8684(A)‐64, the higher density of polar ester and carboxyl groups can promote strong hydrogen bonding. At the same time, LNMO particles, which exhibit metal–oxide surfaces, can engage in hydrogen bonding with the acidic or polar groups of the binder, thus improving both adhesion and mechanical integrity of the electrode [54]. Nevertheless, in a waterborne environment, excessive hydrophilicity can also translate into pronounced binder swelling or partial solubilization, risking film deformation or binder “bleeding” under high‐humidity conditions [55]. These same polar interactions can also occasionally lead to increased interfacial reactivity, specifically under the high‐voltage conditions encountered during battery cycling for LNMO cathodes—either by raising local solvent reorganization energies or by influencing interfacial stability (CEI layers) under high‐voltage operation [56, 57].

In contrast, TRD202A, with its comparatively subdued C─O bands and more prominent methyl C─H stretches, suggests a more hydrophobic nature, which may reduce the extent of hydrogen bonding or ionic interactions at the interface. In this case, the assumption that TRD202A contains fluoropolymer‐derived domains is especially significant. Fluorine‐containing groups, when present, are known for their chemical inertness and low surface energy, which can help minimize side reactions at the binder‐active material interface [58]. Although distinct C─F stretching bands are not clearly defined and evident—implying any fluorinated segments are either present in low concentration or are spectrally masked—these domains could act to shield the polymer matrix from oxidative environments while still permitting sufficient adhesion through the remaining acrylic components.

Ultimately, the nature and balance of these chemical bonds—hydrogen bonds, van der Waals interactions, and any potential ionic or dipole–dipole attractions—can affect key performance indicators such as mechanical robustness, adhesion strength, and electrolyte compatibility. These factors, in turn, can influence the overall electrochemical performance of waterborne LNMO cathode systems. Understanding these differences is essential for interpreting the electrochemical results and drawing final conclusions later in this work.

Together with FTIR analysis, TGA was employed to better understand the thermal stability of each binder, decomposition temperatures, and residual char yield under inert and oxidative atmospheres, thereby providing further insight into their thermal behavior and supporting the distinction between the two binder systems. Thermogravimetric profiles (Figure 2) again underscore the fundamental differences between the two binders chemistries. Under nitrogen (Figure 2a), SX8684(A)‐64 undergoes a single, abrupt mass‐loss event—about 92% of its weight between room temperature and ~410 °C—leaving only ~1.5% char behind, fully consistent with the behavior of high‐molecular‐weight PMMA homopolymers that decompose in one principal step under inert conditions [59, 60, 61]. In different TGA studies conducted on PMMA, a dominant derivative thermogravimetric (DTG) peak appears around 350 °C with negligible secondary shoulders, and char yields under N_2_ rarely exceed 2%, underscoring the SX8684(A)‐64 polymethacrylate backbone purity [62]. Under air (Figure 2b), the oxidative environment shifts its degradation onset to ~425 °Cbut still collapses in a single major event (≈90% weight loss), as SX8684(A)‐64 combusts cleanly, leaving <1% residue, a hallmark of simple ester‐rich polymers [63, 64]. By contrast, TRD202A undergoes multistage decomposition in nitrogen (Figure 2c)—initially losing ≈76% below 420 °C, then shedding another ~4% at 420–440 °C, ~9% at 440–475 °C, and ~5% by 800 °C—mirroring the behavior of hybrid acrylic/fluoropolymer latex systems, where a primary low‐temperature loss corresponds to acrylic chain scission and higher‐temperature events map to thermally robust fluorinated domains [65]. Such staggered degradation is frequently reported for copolymer latex blends of methyl methacrylate with acrylate monomers, which display multiple DTG peaks correlating to discrete backbone and side‐chain cleavage [66, 67, 68]. In air (Figure 2d), TRD202A again shows a multipeak profile—~74% loss to 410 °C, ~16% to 475 °C, and ~9% to 800 °C—with essentially zero residual char (<0.3%), reflecting eventual combustion of its fluoropolymer fractions once oxygen is present [69]. By comparison, pure PVdF binders typically yield ~20%–30% char under inert conditions before combusting fully in air, reflecting the high thermal stability of fluoropolymer backbones [70].

**FIGURE 2:**
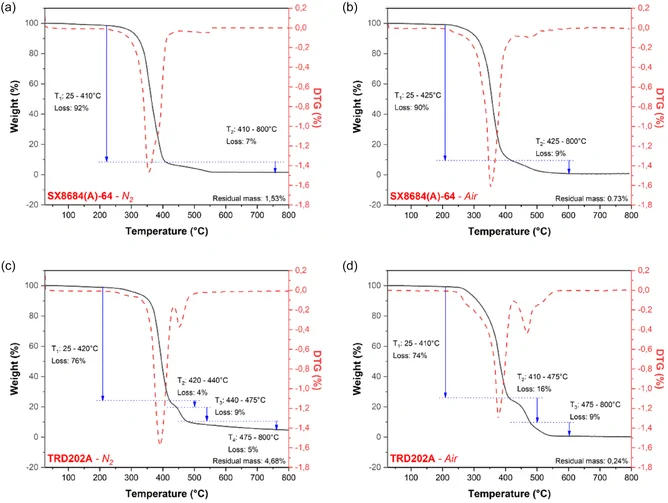
TGA (solid lines) and derivative mass loss (dashed lines) of SX8684(A)‐64 and TRD202A binders under (a,c) nitrogen and (b,d) air atmospheres.

Collectively, these thermograms reinforce the previous FTIR inference of SX8684(A)‐64 as a single‐phase polymethacrylate and TRD202A as a composite latex: the former cleanly volatilizes in one step, while the latter's multistep, higher‐temperature weight losses and significant inert‐atmosphere char are consistent with a hybrid architecture comprising acrylic and potentially fluorinated components [71, 72].

After establishing the chemical structures and composition of both binders, their interaction with the LP40 electrolyte (1 M LiPF_6_ in EC:DEC 1:1 v/v) was evaluated. Figure S1 illustrates the swelling‐test disks before and after 24 h of immersion in the electrolyte. Visually, both disks retain their original color, with only a subtle increase in opacity evident after soaking. Likewise, any changes in disk dimensions are minimal and become apparent only upon close measurement rather than by a casual, visual inspection. Table 1 summarizes the measured volumetric and mass increases for each sample, along with the calculated swelling ratios. All measurements were replicated three times to ensure statistical confidence.

**TABLE 1:** Mass‐ and volume‐based swelling of SX8684(A)‐64 and TRD202A binder disks after 24 h in LP40 electrolyte. Each sample weight and volume increase is reported as a percentage of its dry state, revealing minimal swelling (<3% by mass, <7% by volume) for SX8684(A)‐64 versus significantly greater uptake for TRD202A (≈33% mass, ≈20% volume).

Sample	Mass based swelling			Volumetric swelling		
	*W*_dry_, mg	*W*_swollen_, mg	Swelling ratio, %	*V*_dry_ , mm^3^	*V*_swollen_, mm^3^	Swelling ratio, %
SX8684(A)‐64—1	449	461	2.67	555	592	6.72
SX8684(A)‐64—2	569	581	2.11	736	778	5.67
SX8684(A)‐64—3	553	566	2.35	822	864	5.07
TRD202A—1	373	500	34.05	334	393	17.65
TRD202A—2	373	495	32.71	385	464	20.54
TRD202A—3	383	515	34.46	406	486	19.71

The limited swelling of SX8684(A)‐64, just 2%–3% mass uptake and <7% volumetric expansion, is fully in line with polymethacrylate binders, which typically absorb less than 5% electrolyte, preserving mechanical stiffness and ensuring stable film integrity under cycling [73]. Conversely, binders with insufficient electrolyte affinity (<2% swelling) can leave pore networks poorly wetted, elevating ionic‐transport resistance and degrading power performance [74].

By contrast, TRD202As substantial uptake, over 30% by mass and ~20% by volume, reflects its hybrid acrylic/fluoropolymer design that leverages controlled gel‐like swelling to create continuous Li^+^‐permeable pathways throughout the electrode [75]. Such swelling‐mediated plasticization has been directly linked to lower ionic tortuosity and enhanced fast‐charge capability in LiBs, where expanded binder networks reduce diffusion path lengths and improve interparticle ionic conduction [16, 73]. The multistage TGA decomposition, together with FTIR results, is consistent with the presence of fluorinated segments, which may contribute to mechanical reinforcement, potentially mitigating film softening even at high electrolyte uptake and preventing the delamination issues seen in overly swollen, noncross‐linked systems [76].

After the swelling test, a CV test on both binder samples was carried out to assess their electrochemical stability and redox activity and to detect any swelling‐induced degradation or parasitic reactions that could compromise cathode performance at the cell level. The electrochemical stability of both binder samples is presented in Figure S2. Both the full‐range and zoomed‐in sections of the CV curves reveal only minor current responses in the anodic and cathodic sweeps—on the order of microamperes—with no noticeable redox peaks. These minimal current variations, consistent with literature reports for similar binder systems [77, 78], indicate that both binders remain electrochemically stable within the operational voltage window for LNMO (3–4.9 V).

Instead, Figure 3 reveals clear distinctions in the redox behavior and reversibility of cathodes manufactured using SX8684(A)‐64 versus TRD202A—and in how bare versus carbon‐coated aluminum collectors influence those reactions. In the SX8684(A)‐64 panels, the bare‐foil cell (Figure 3a) shows broad, low current peaks for both the Mn^4+^/Mn^3+^ couple (~3.8 V) and the Ni^2+^/Ni^3+^ and Ni^3+^/Ni^4+^ plateaus (~4.7–5.0 V), with significant peak‐separation growth from cycle 1–5—evidence of sluggish charge transfer and growing polarization, an indication of heightened interfacial resistance possibly due to foil corrosion and/or poor adhesion—effects that have been directly linked to uncoated current collectors in LNMO cells [15, 79]. When SX8684(A)‐64 is coated onto carbon‐treated aluminum (Figure ‍3b), both peak sharpness and peak‐current density improve by roughly 20%–30%, reflecting the enhanced electronic contact and corrosion suppression provided by the carbon interlayer—a result in excellent agreement with studies showing over 30% boost in LNMO rate performance and CV peak definition on carbon‐coated collectors [15, 80, 81].

**FIGURE 3:**
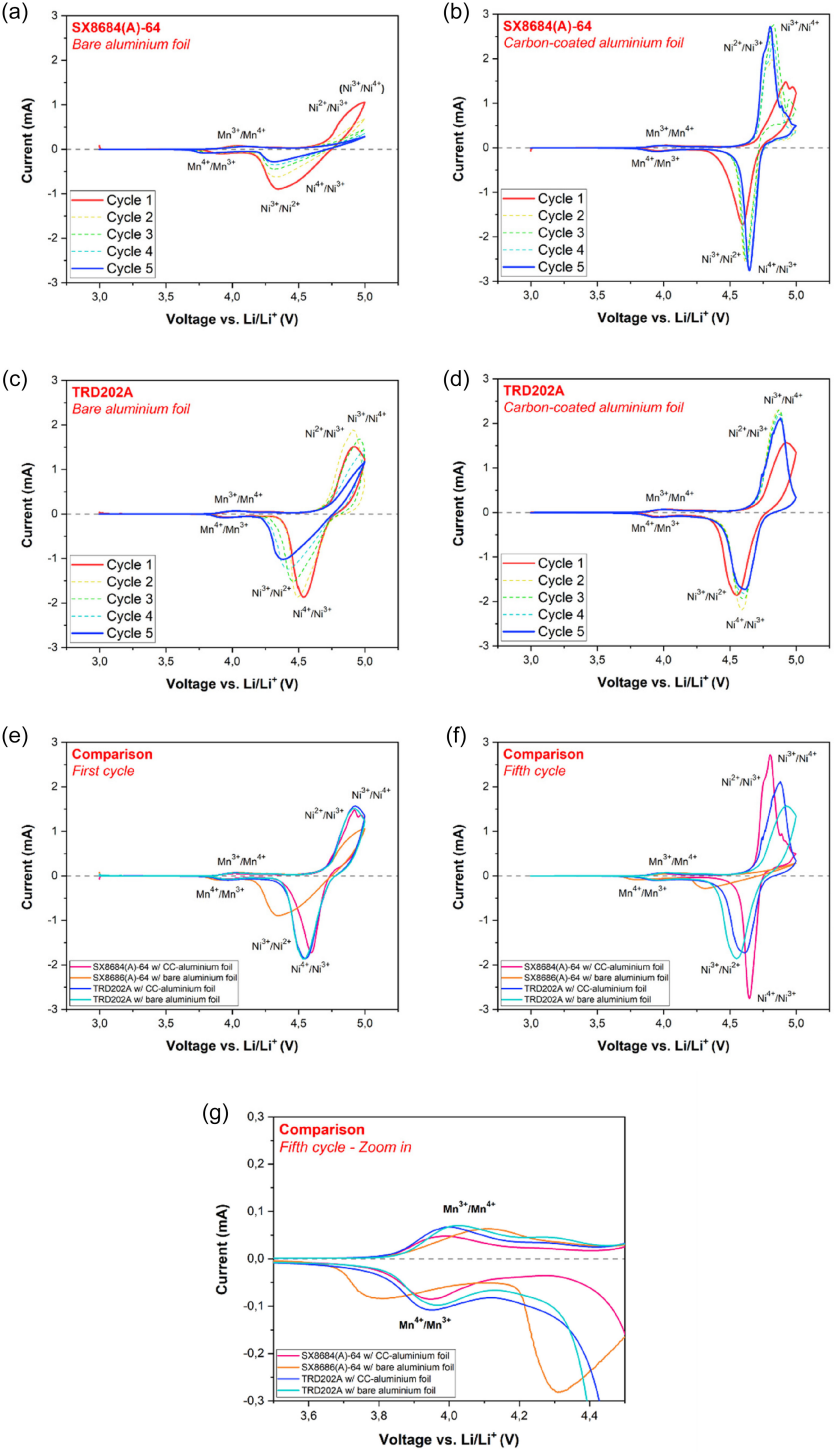
CV traces of LNMO electrodes with SX8684(A)‐64 (a,b) and TRD202A (c,d) binders on bare and carbon‐coated aluminum foil over the first five cycles at 0.1 mV s^−1^. Panels (e,f) overlay the first and fifth cycles for all four configurations, highlighting improvements in peak shape and reduced polarization for TRD202A on carbon‐coated foil. Panel (g) provides a zoomed view of the Mn^3+^/Mn^4+^ redox region on the fifth cycle, illustrating the sharper, more symmetric peaks achieved with the TRD202A‐CCAF pairing.

Electrodes formulated with the hybrid TRD202A binder exhibit more symmetric redox profiles even on bare Al (Figure 3c, both Mn and Ni peaks already overlap closely from cycle 2 onward), as the binder network and improved interfacial contact promote more efficient charge‐transfer kinetics and mitigate polarization. A similar behavior was observed for sulfated alginate, thiophene‐derived polymers, and other similar hybrid binders, which reduced interfacial resistance and produced sharper CV peaks in high‐voltage spinel cathodes [82, 83]. The combination of TRD202A with carbon‐coated foil (Figure 3d) delivers the most pronounced enhancements: Ni and Mn peaks maintain nearly constant anodic and cathodic currents across five cycles, with Δ*E*_p_ below 100 mV at the Ni^3+^/Ni^4+^ transition, suggesting improved interfacial stability, consistent with previous work findings [84], as well as industrial reports of LNMO cells on protective carbon‐Al collectors, which observed superior capacity retention and overlapping CV profiles [85].

Overlay comparisons on the first and fifth cycles (Figure 3e,f) clearly show that the TRD202A + carbon‐coated aluminum foil configuration maintains the best overall reversibility, while SX8684(A)‐64—even on coated foil—exhibits progressive polarization, echoing findings in other LNMO studies where binder‐collector interface engineering was critical for sustaining sharp CV profiles at high voltage [86, 87]. The zoom‐in of the Mn^3+^/Mn^4+^ couple (Figure 3g) highlights how only the TRD202A + carbon‐coated aluminum foil configuration sustains strong, symmetric Mn redox peaks at ~3.8 V with negligible shift.

Together, these results align with literature demonstrating that carbon‐coated aluminum collectors not only protect the foil from hydrofluoric acid attack and mitigate corrosion, but also synergize with conductive binder networks to minimize charge‐transfer resistance and preserve high‐voltage redox kinetics in water‐processed LNMO cathodes. Such synergy between corrosion‐resistant carbon‐coated current collectors and advanced binder chemistries has been shown to lower contact resistance by tens of ohm–cm^2^, improve overall electrode kinetics, and sustain high *C*‐rate performance in water‐processed LNMO cathodes [15, 79]. These observations are further reinforced by waterborne LNMO formulations tested in pouch cells, where aqueous binder systems matched or exceeded PVdF‐NMP characteristics when paired with carbon‐coated collectors [88] and by high‐mass‐loading LNMO electrodes that retained sharp, high‐current CV profiles only on carbon‐coated substrates under aggressive cycling [38]. Moreover, carboxymethyl cellulose‐based binders—traditionally less conductive —have been shown to yield comparable Mn and Ni peak symmetry and improved de‐lithiation kinetics when optimized in hybrid systems, underscoring the critical role of binder‐collector interplay in unlocking durable, low‐impedance interfaces for high‐voltage LiNi_0.__5_Mn_1.__5_O_4_ cathodes [89].

To further investigate how the conductive carbon‐coating synergizes with each binder chemistry, we employed ICI to extract state‐of‐charge (SOC)‐dependent ohmic and charge‐transfer resistances during galvanostatic cycling (in terms of cell ohmic resistance (*R*_0_) and apparent charge‐transfer resistance (*R*_ct_) at different SOC values). Following the approach used in previous works, the instantaneous voltage drop at 0.1 s can be directly correlated with *R*_0_, while the total drop at 2 s encompasses both *R*_0_ and *R*_ct_, from which *R*_ct_ can be obtained by subtracting the previously determined *R*_0_ [44, 45, 90, 91]. The resulting graphs can be seen in Figure 4, in which both SOC‐dependent *R*_0_ and *R*_ct_ values obtained during charging via ICI are plotted, comparing electrodes manufactured using both SX8684(A)‐64 and TRD202A binders on bare and carbon‐coated aluminum foil immediately after formation cycling and after 100 full cycles. In Figure 4a,c, *R*_0_ remains below 5 Ω between ~10% and 90% SOC, but steeply rises near 0% and 100% SOC, possibly reflecting increased polarization at the electrode/electrolyte interface at the start and at the end of the charging cycle [92, 93]. Interestingly, the bare Al foil exhibits slightly lower *R*_0_ values than CCAF near the SOC extremes, suggesting that the carbon coating does not reduce the purely ohmic contribution under these conditions. In contrast, the *R*_ct_ trend (Figure 4b,d), albeit similar to the *R*_0_ ones, reveals a clear minimum plateau within 10%–90% SOC, with less noticeable and pronounced minima in the 40%–60% region, where interfacial charge‐transfer kinetics are most favorable [94]. Here, the combination of the TRD202A binder and carbon coating yields the lowest *R*_ct_ values (≈50 Ω post‐formation; ≲70 Ω after 100 cycles), reflecting enhanced ionic/electronic pathways offered by conductive polymeric binders and robust electrode‐foil adhesion [95, 96]. By contrast, SX8684(A)‐64 on bare foil exhibits persistently elevated *R*_ct_ (≈350 Ω post‐formation; >500 Ω after cycling), consistent with the buildup of resistive interphases observed for less conductive binders. Even after 100 cycles, the *R*_0_ and *R*_ct_ profiles maintain the same trend: TRD202A + coated‐foil cells show minimal resistance growth, while bare‐foil and SX8684(A)‐64‐based cells suffer significant increases, highlighting the stability of the optimized interface [94]. Insets magnify the sub‐10% SOC region, where TRD202A formulations—regardless of the foil type—suppress the initial resistance rise more effectively than SX8684(A). Overall, even after extensive cycling, the combination of the TRD202A binder and CCAF delivers the most stable, low‐resistance interface, minimizing both ohmic and kinetic barriers throughout the full charge process. These findings align with binder‐design principles that promote effective interfacial charge transport and strong interfacial adhesion to minimize both ohmic and kinetic barriers during aggressive cycling [97].

**FIGURE 4:**
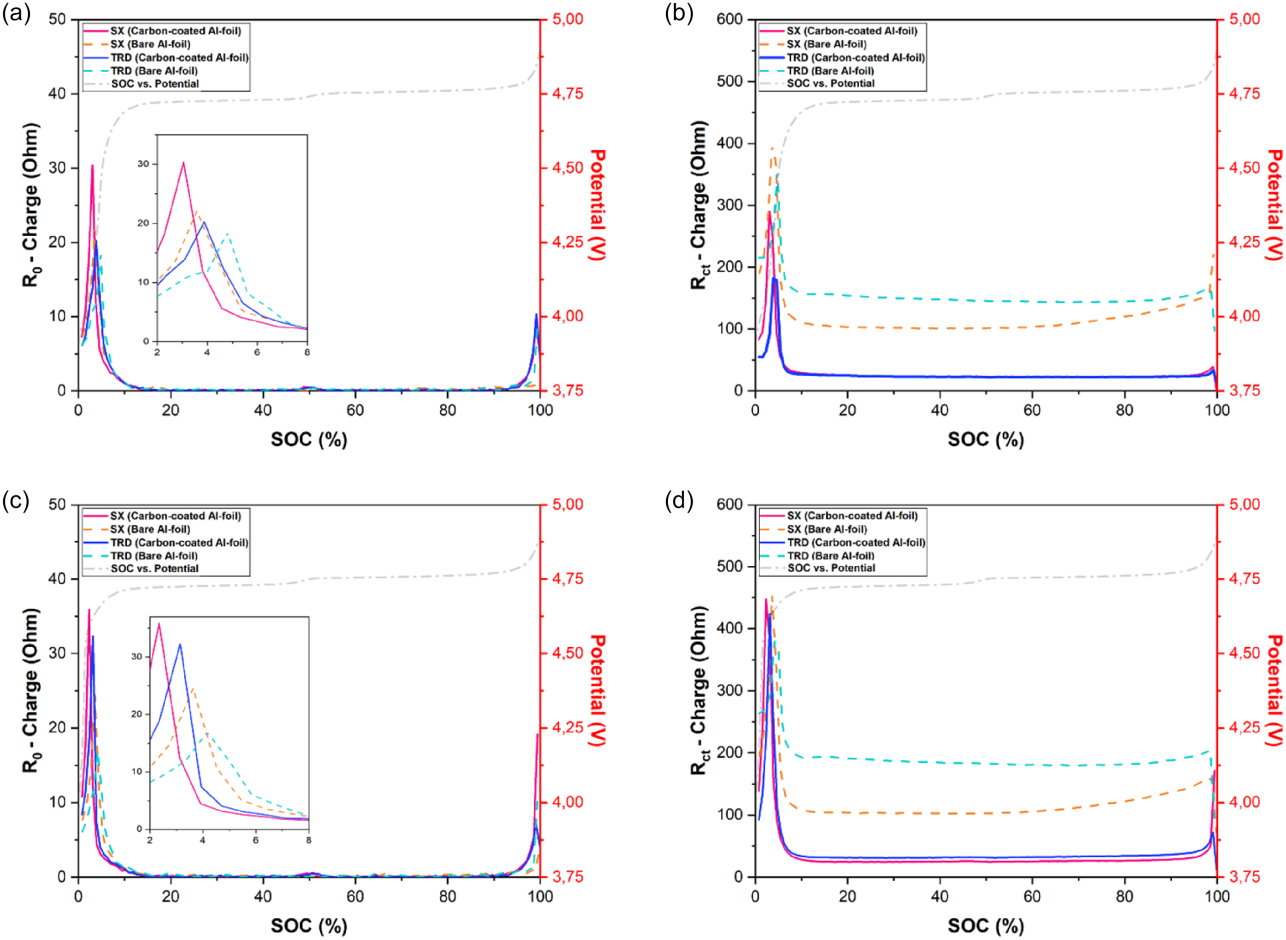
SOC‐dependent resistances extracted by ICI during charging for LNMO electrodes with SX8684(A)‐64 and TRD202A binders on bare and carbon‐coated aluminum: (a) *R*_0_ after forming cycles, (b) *R*_ct_ after forming cycles, (c) *R*_0_ after 100 cycles, and (d) *R*_ct_ after 100 cycles. Insets zoom into the low‐SOC region to highlight the suppressed resistance rises achieved by the TRD202A/carbon‐coated foil configuration.

Before proceeding with the galvanostatic cycling and the evaluation of the electrochemical performance of each sample, morphological analysis via FESEM (Figure 5) was carried out to find structural, larger‐scale evidence of the compatibility between each current collector and the two different cathodic slurries. Across all magnifications, the carbon‐coated coated‐foil electrodes (Figure 5a,b,e,f,i,j,m,n) display a more uniform, crack‐free microstructure than their bare‐foil counterparts (Figure 5c,d,g,h,k,l,o,p). At the lowest magnification (500×), the SX8684(A)‐64 and TRD202A cathode coatings (Figure 5a,b) on carbon‐treated foil form a continuous layer with no visible delamination or large fissures, whereas both bare‐foil samples (Figure 5c,d) already show the beginnings of microcracks. Moving to 1000×, the LNMO particles in the carbon‐coated aluminum foil electrodes (Figure 5e,f) remain embedded in the polymer matrix, with only small, evenly distributed pores; in contrast, the bare‐foil electrodes (Figure 5g,h) exhibit larger, irregular voids and patches where the active material appears to pull away from the aluminum.

**FIGURE 5:**
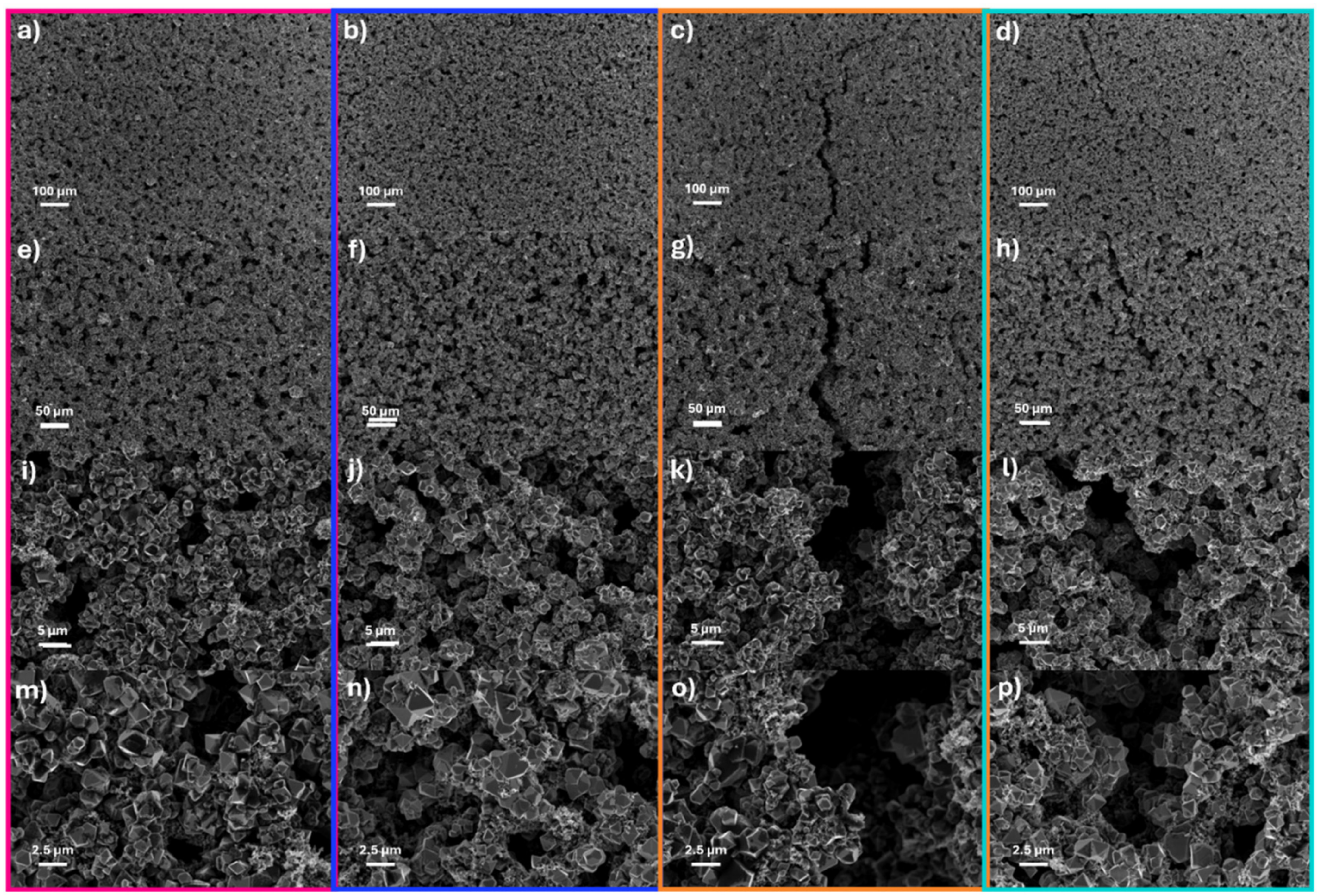
Cross‐sectional FESEM micrographs of LNMO cathodes coated with SX8684(A)‐64, at increasing magnifications. SX8684(A)‐64 on carbon‐coated Al foil at: (a) 500×, (e) 1k×, (i) 5k×, and (m) 10k×. TRD202A on carbon‐coated Al foil at: (b) 500×, (f) 1k×, (j) 5k×, and (n) 10k×. SX8684(A)‐64 on bare Al foil at: (c) 500×, (g) 1k×, (k) 5k×, (o) and 10k×. TRD202A on bare Al foil at: (d) 500×, (h) 1k×, (l) 5k×, and (p) 10k×.

At 5000× magnification, the carbon‐coated foil electrodes (Figure5i,j) display a more uniform particle packing with smaller, evenly distributed pores, while the bare‐foil counterparts (Figure 5k,l) show larger, irregular voids and initial fissuring between clusters of LNMO grains. At 10 000×, the carbon‐coated samples (Figure 5m,n) maintain this homogeneous, tightly packed granular network, whereas the bare‐foil coatings (Figure 5o,p) exhibit widened intergranular gaps and pronounced microcracks that penetrate into the electrode layer. These observations suggest that the carbon interlayer promotes a more cohesive and crack‐resistant microstructure.

In summary, the FESEM series demonstrates that carbon‐coated current collectors markedly improve coating integrity and that the TRD202A binder further enhances particle cohesion and interfacial adhesion— minimizing voids, microcracks, and delamination even at the sub‐micron level.

These FESEM observations closely align with several recent studies on the LNMO cathode microstructure and current‐collector engineering. Kuenzel et al. demonstrated that carbon‐coated Al foils produce crack‐free, tightly adhered LNMO films, whereas bare Al substrates exhibit microfissures and delamination under identical coating and cycling conditions [38, 80]. The improvement stems from functional carbon groups on the foil that cross‐link with the binder matrix, thereby reducing interfacial resistance and preventing crack initiation [15]. Binder surface free energy has also been shown to govern polymer spreading and particle encapsulation: in a recent study, Weber et al. found that tailoring binder polarity yields more uniform coverage and fewer voids in LNMO electrodes, as evidenced by SEM [98, 99]. In comparisons of PAN versus PVdF‐hexafluoropropylene binders, Mathew et al. demonstrated how polar PAN formulations can achieve homogeneous carbon black and binder distribution around LNMO particles—mirroring our high‐magnification images of tight, crack‐free interfaces—while PVdF‐based electrodes suffered uneven additive dispersion and early microcracking [92]. Composite electrodes engineered with enhanced adhesive forces likewise exhibited stronger binder bridges and minimal pore formation in FESEM cross‐sections, consistent with our observations [100]. Analogous findings in aqueous supercapacitor studies show that graphite passivation layers eliminate interfacial voids and uphold electrode integrity under high magnification [101]. Collectively, these studies corroborate that carbon‐coated collectors and optimized binder chemistries synergistically produce the highly uniform, crack‐free microstructures revealed in our FESEM series. More generally, it is worth noting that macro‐ and micromorphological control has been recognized as a key determinant of conductivity and cycling stability in 5 V spinel cathodes [102].

To better analyze the role of different binders combined with different current collector surfaces, porosity and tortuosity were quantified via 2D image analysis of cross‐sectional FESEM micrographs, following the workflow proposed by Rabbani et al. [46, 47]. Figure S3 schematizes the multistep, script‐based procedure comprising image preprocessing, pore‐thresholding, and porosity calculation, while Figure S4 illustrates a representative output showing pore masks overlaid on raw FESEM data. Indeed, the use of image‐based segmentation, following established FESEM protocols for lithium‐ion electrodes, provides direct visualization of pore networks and yields tortuosity estimates comparable to those derived from impedance‐spectroscopy methods [103, 104, 105].

For each electrode, five different areas were analyzed to account for microstructural heterogeneities, and the reported porosity is the average of these five measurements [105]. To validate the image‐derived values, porosity was also mathematically calculated using the ratio of measured mass loading to the product of electrode thickness and composite theoretical density [106] and the bulk/theoretical density formalism commonly applied in electrode modeling, as reported in Equations (1) and (2):

(1) $$\varepsilon =1-\frac{{\mathrm{ML}}_{\mathrm{coat}}}{L\rho_{\mathrm{th}}}$$

(2) $$\rho_{\mathrm{th}}≅\frac{100}{\sum (x_{i}/\rho_{i})}$$

In this expression, *ε* denotes the electrode porosity, ML_coat_ is the mass loading of the electrode encompassing both active and inactive materials per unit area, *L* is the coating thickness of the current‐collector foil, and *ρ*_th_ is the composite theoretical (skeletal) density, computed as the sum of each constituent true density *ρ* *_i_* weighted by its mass fraction *x* *_i_*. Skeletal densities were obtained from supplier datasheets or the literature, respectively: 4.2 g cm^−3^ for LiNi_0.__5_Mn_1.__5_O_4_, 1.6 g cm^−3^ for carbon black C45, and 1.6 g cm^−3^ for NaCMC. For the polymer binders, we estimated true densities based on their chemical classes: SX8684(A)‐64 was approximated as the mean of PMMA (1.19 g cm^−3^) and SBR (0.94 g cm^−3^), yielding 1.065 g cm^−3^, while TRD202A was taken as the midpoint of acrylic latex (~1.19 g cm^−3^) and PVdF (1.78 g cm^−3^), giving ~1.485 g cm^−3^. The porosities calculated via this method are presented alongside image analysis results in Table 2.

**TABLE 2:** Porosity, average pore radius, and tortuosity determined by FESEM micrograph analysis, alongside mass‐loading/thickness modeling results for SX8684(A)‐64 and TRD202A binders on bare and carbon‐coated aluminum foil.

Sample	Obtained via imaging			Obtained via modeling			
	Porosity, %	Av. pore radius, µm	Tortuosity	ML_coat_, mg cm^−2^	*L*, µm	*ρ*_th_ , g cm^−3^	Porosity, %
SX8684(A)‐64 w/bare AF	66.9	4.40	3.71	14.50	110.96	3.55	63.2
SX8684(A)‐64 w/CCAF	69.7	4.43	2.64	10.63	92.50	3.55	67.6
TRD202A w/bare AF	67.1	3.95	2.62	13.73	114.27	3.69	67.6
TRD202A w/CCAF	69.4	4.25	2.38	12.80	104.27	3.69	66.7

From the FESEM‐based analysis, SX8684(A)‐64 on bare Al exhibits the highest tortuosity (*τ* ≈ 3.7) and the second largest average pore radius (≈4.4 µm) at a porosity of ~67%, reflecting the poorly adhered, crack‐prone coatings typical of untreated foil interfaces [107]. Switching to a carbon‐coated collector cuts tortuosity by ~30% (*τ* ≈ 2.6) with only a minor drop in porosity, in agreement with reports that carbon‐coated aluminum foils dramatically reduce contact resistance and foster more uniform, crack‐free films [15, 108]. The TRD202A‐based films start out less tortuous than their SX counterparts on bare foil (*τ* ≈ 2.6 vs. 3.7) and lose up to ~9% more tortuosity when cast on coated foil (down to *τ* ≈ 2.4), while maintaining ~69% porosity and slightly smaller pores, consistent with hybrid acrylic/fluoropolymer chemistries that promote tighter particle‐binder cohesion and stable pore architectures in high‐voltage spinels [109].

The modeling approach—using measured mass loading, coating thickness, and the composite theoretical density—recovers the same relative ranking: SX8684(A)‐64  +  bare Al has the lowest modeled porosity (≈63%), consistent with the highest tortuosity obtained from image analysis; SX8684(A)‐64  +  CCAF and both TRD202A configurations cluster around 67% porosity.

Although the absolute porosity values differ by up to ~4 percentage points (imaging yields 67%–70% and modeling 63%–68%), both methods converge on similar conclusions. The close agreement between the two methods (all imaging‐derived porosities lie within ~4% of their modeled counterparts) underscores the robustness of our analysis and validates the robustness of combining gravimetric and image‐based methods for porosity estimation [103]. Importantly, the close agreement between imaging and modeling underscores that both techniques capture the underlying microstructural trends, from the densest SX8684(A)‐backed coatings on bare foil to the most open, least tortuous TRD202A films on carbon‐coated collectors [79, 81]. From an electrochemical perspective, the lower tortuosity and high porosity of the TRD202A on the carbon‐coated aluminum foil current collector configuration suggest more favourableion‐transport pathways, aligning with its superior rate capability and reduced *R*_ct_ observed in ICI tests.

Finally, starting from the results collected through the previous chemo‐physical and morphological analyses, we evaluated the electrochemical performance of all four electrode configurations by galvanostatic cycling using the protocols and methods already indicated in the Experimental Section. Figure 6 illustrates the rate‐capability performance of LNMO electrodes prepared with the two binder chemistries (SX8684(A)‐64 vs. TRD202A) and two types of aluminum current collectors (bare vs. carbon‐coated). Across a sequence of charge/discharge rates—from C/10 and C/5 symmetrical charge/discharge to 1 C charge/5, 10, and 15 C discharge and back to C/10 symmetrical cycling—the combination of the TRD202A binder with carbon‐coated foil consistently delivers the highest capacities and capacity retention. At low rates (C/10 and C/5), all four‐electrode configurations delivered ≈130 mAh g^−1^ with minimal variation. Increasing the C‐rate to 1 C (cycles 9–12), the TRD202A‐based electrodes retain ≈120 mAh g^−1^ specific discharge capacity on carbon‐coated aluminum foil and ≈115 mAh g^−1^ specific discharge capacity on bare aluminum foil, outperforming the SX8684(A)‐64‐based samples (≈115 mAh g^−1^ coated ≈100 mAh g^−1^ bare). This behavior can be attributed to enhanced ionic/electronic pathways imparted by the different chemistries of the binders, as also reported for other binder systems [13]. Under more stressful discharge conditions (1 C/5 C, 1 C/10 C, and 1 C/15 C), only electrodes on carbon‐coated collectors sustain appreciable capacity: TRD202A + CC‐coated delivers 100–110 mAh g^−1^ at 5 C, 60–70 mAh g^−1^ at 10 C, and 45–50 mAh g^−1^ at 15 C, whereas SX8684(A)‐64 + CC‐coated falls to 85–95 mAh g^−1^ (5 C) and 30–45 mAh g^−1^ (10–15 C), confirming that carbon coatings on aluminum collectors can help to substantially reduce contact resistance and enhancing rate capability [81]. As mentioned before, the carbon layer coated on the CC also provides functional groups that potentially cross‐link the electrode coating to the foil, stabilizing the interface during fast cycling [80]. In contrast, bare‐foil cells collapse to near zero capacity above 5 C, consistent with the observation reported in the literature, highlighting the increase in polarization resistance when high *C*‐rates are applied on electrodes with untreated collectors [110]. Upon returning to C/10 in cycle 28, the coated‐Al foil electrodes recover nearly all of their initial capacities (TRD ≈ 130 mAh g^−1^; SX ≈ 125 mAh g^−1^), whereas bare‐foil samples only rebound to ≈110−108 mAh g^−1^; this excellent reversibility on coated foil aligns with studies on both LNMO and other high‐voltage materials showing full capacity recovery after high‐rate cycling [111]. These capacity trends are further confirmed by the bar‐plot data in Figure 6, which compares the average discharge capacities of SX8684(A)‐64 and TRD202A electrodes across the same *C*‐rate protocol, including the last 100 cycles performed at a symmetrical C‐rate of 1 C.

**FIGURE 6:**
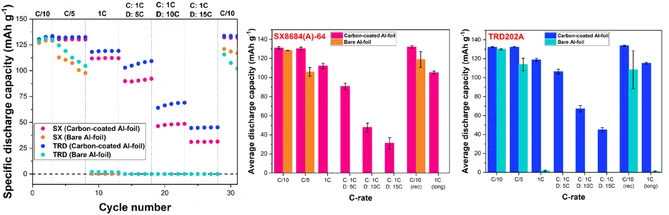
Galvanostatic rate‐capability profiles and bar diagram comparison of LNMO cathodes using SX8684(A)‐64 and TRD202A binders on bare and carbon‐coated aluminum foil. Specific discharge capacities are plotted versus cycle number across a protocol of C/10  →  C/5  →  1 C charge/5 C  →  1 C/10 C  →  1 C/15 C discharge, returning to C/10. In the bar diagram, the average discharge capacity value relative to the last 100 cycles at 1 C is also reported.

Since the carbon‐coated current collector showed a significant influence on the electrochemical properties of the electrode, it was selected for the final study and comparison between the two investigated binders: SX8684(A)‐64 and TRD202A. Figure 7 compares the charge–discharge profiles of SX8684(A)‐64 and TRD202A cathodic formulations on CCAF at three points in their cycling life: the initial cycle, post‐forming (cycle 8), and after 100 cycles at 1 C. In the first forming cycle, both electrodes display the characteristic two‐plateau LNMO behavior at ~4.0 V (Mn^3+^/Mn^4+^) and ~4.7 V (Ni^2+^/Ni^3+^ at 4.65 V and Ni^3+^/Ni^4+^ at 4.75 V), delivering nearly identical specific capacities of ~135 mAh g^−1^ with minimal overpotential (voltage gap <0.1 V) [112, 113]. By cycle 8, however, the TRD202A‐based electrode shows lower polarization and slightly higher discharge capacity (~130 mAh g^−1^ versus ~125 mAh g^−1^ for SX8684(A)‐64), reflecting enhanced interfacial stability [114]. After 100 cycles, the SX8684(A)‐64 cell loses a substantial fraction of its high‐voltage plateau, delivering just 100 mAh g^−1^ and exhibiting a broadened voltage hysteresis, consistent with growing cell polarization [115]. In contrast, the TRD202A electrode sustains ~120 mAh g^−1^ with only modest plateau contraction and a narrow hysteresis loop, suggesting comparatively better preservation of the cathode‐side interface and conductive network [113, 114]. It should be noted that, in the half‐cell configuration employed here, increases in cell impedance over long‐term cycling may partly reflect changes at the lithium counter/reference electrode rather than exclusively at the cathode [116]. The relative trend between the two binder systems is nonetheless consistent across all electrodes, which were cycled under identical conditions against fresh lithium counter‐electrodes.

**FIGURE 7:**
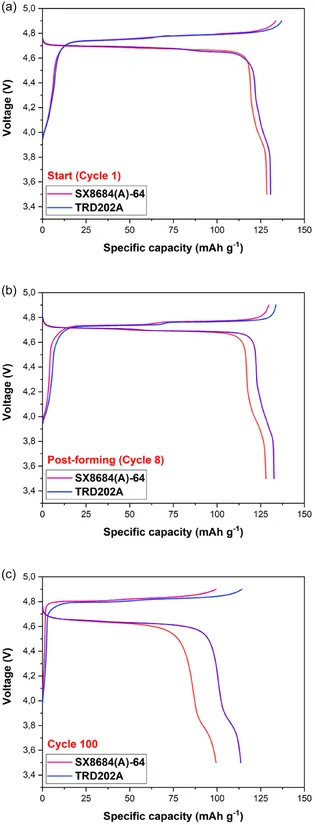
Charge–discharge voltage profiles of LNMO cathodes with SX8684(A)‐64 and TRD202A binders on carbon‐coated aluminum foil at C/10 for (a) the first cycle, (b) after formation (cycle 8), and (c) after 100 cycles at 1 C.

These trends mirror findings in high‐voltage systems utilizing functional binders like poly(3,4‐ethylenedioxythiophene):poly(styrenesulfonate) and sulfated alginate, which achieve capacity retentions above 90% after 100–200 cycles by reinforcing electronic/ionic pathways and stabilizing electrode–electrolyte interfaces [83, 117]. Collectively, these profiles underscore that the TRD202A binder, in concert with a carbon‐coated current collector, robustly mitigates impedance growth and structural degradation, delivering the best capacity retention and lower overpotential in LNMO cathodes.

As a final comparison, the galvanostatic cycling graph relative to the final 100 cycles at 1 C for the two most promising systems, SX8684(A)‐64 and TRD202A on carbon‐coated Al foil, is reported in Figure 8. In the final 100‐cycle segment at 1 C, both binder systems exhibit impressive reversibility, but the TRD202A formulation stands out: while SX8684(A)‐64 declines from ~112 to ~98 mAh g^−1^ over 100 cycles (≈87.5% capacity retention), TRD202A goes from ~120 to ~108 mAh g^−1^, showing a substantial improvement both in terms of raw and percentage‐wise capacity retention (≈90%). This superior cyclability under prolonged 1 C operation reflects the lower tortuosity, higher porosity, and reduced interfacial resistance of the TRD202A/carbon‐coated foil electrode—features that, together, sustain stable Li^+^ conduction pathways and minimize polarization drift over extended cycling.

**FIGURE 8:**
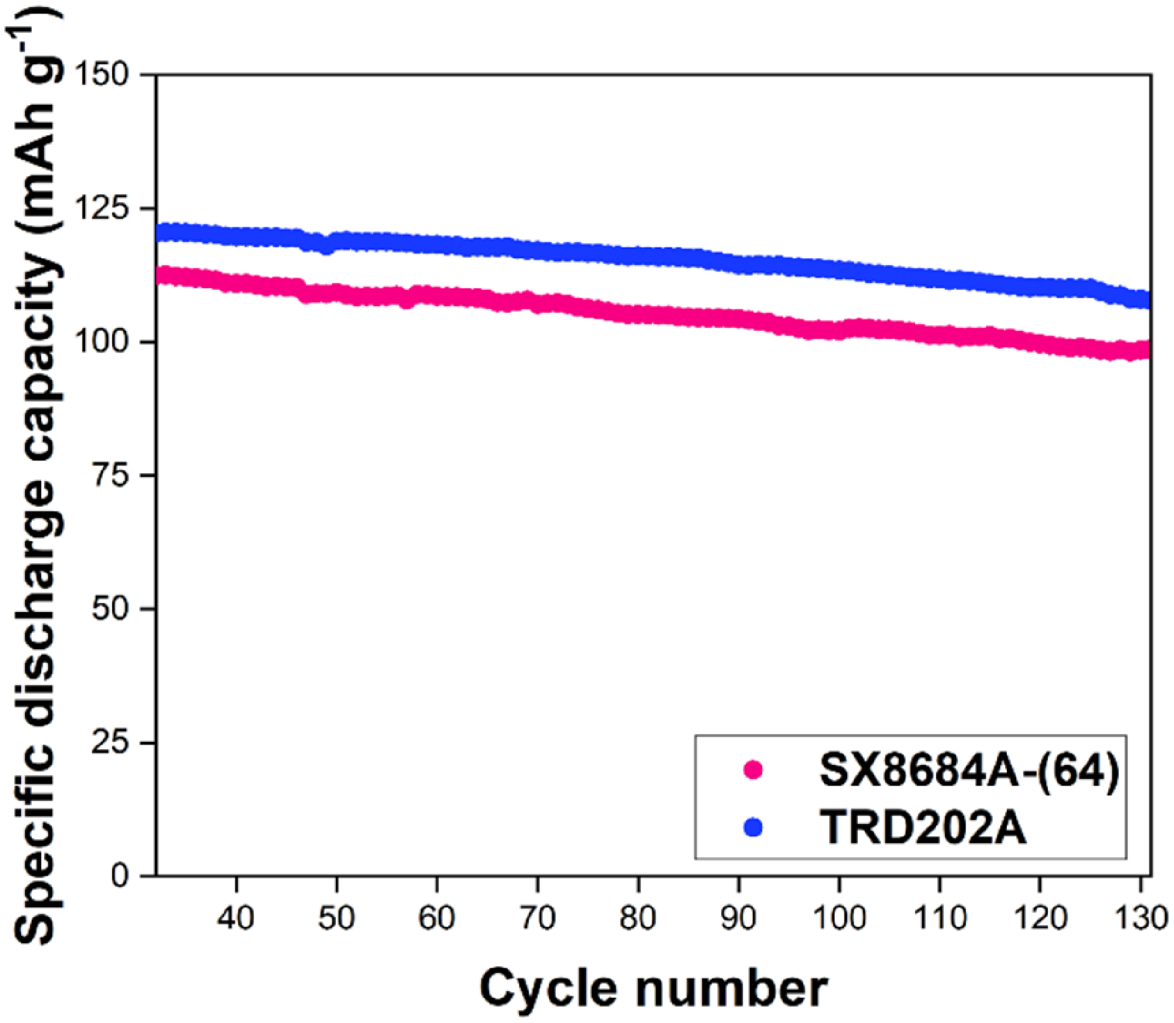
Last 100 cycles specific discharge capacity comparison of LNMO cathodes with SX8684(A)‐64 (pink) and TRD202A (blue) binders on CCAF.

The rate and cycling performance achieved with the TRD202A/CCAF configuration compare favorably with those of other aqueous binder systems reported for LNMO cathodes. Chang et al. demonstrated that a carrageenan‐based binder markedly outperforms both conventional PVdF and sodium alginate in terms of cycling and rate capability on LNMO [28], while Hitomi et‍ al. reported an acrylic‐rubber/CMC composite binder delivering 138 mAh g^−1^ at the first cycle and 128 mAh g^−1^ after 50 cycles, again exceeding PVdF‐based benchmarks [31]. These results, together with the present findings, reinforce the emerging consensus that appropriately engineered aqueous binder chemistries can match or surpass PVdF/NMP performance on high‐voltage LNMO cathodes while eliminating the associated processing and environmental burden.

## 4 Conclusions

In this work, we have demonstrated that the interplay between binder chemistry and current‐collector surface engineering exerts a decisive influence on the performance of aqueous‐processed LNMO cathodes. SX8684(A)‐64, with its uniform polymethacrylate backbone, delivers a dimensionally stable, low‐swelling network that resists electrolyte uptake and maintains mechanical rigidity, yet its limited ionic pathways lead to growing polarization and modest rate capability—particularly on bare aluminum. By contrast, TRD202A hybrid acrylic/fluoropolymer architecture couples substantial, gel‐like swelling with multistep thermal resilience, forming expansive Li^+^‐conductive channels, while potential fluorinated domains may contribute to film cohesion. When paired with carbon‐coated aluminum foils, TRD202A electrodes exhibit the lowest ohmic and charge‐transfer resistances across the whole SOC window, the most uniform, crack‐free microstructure, and the highest capacity retention and electrochemical performance, delivering approximately 45–50 mAh g^−1^ at 15 C and recovering most of their initial capacity upon returning to C/10. Importantly, the implications of this work extend beyond laboratory‐scale optimization. The demonstrated compatibility between aqueous binders and carbon‐coated current collectors at practical mass loadings (10–12 mg cm^−2^) directly addresses one of the most critical bottlenecks in transitioning high‐voltage cathodes away from organic solvent‐based manufacturing. By simultaneously lowering impedance growth, preserving structural integrity, and sustaining high‐rate capability up to 15 C, the optimized TRD202A/CCAF configuration provides a promising basis for further development of scalable aqueous LNMO electrode manufacturing. Such integration of green processing with high‐voltage stability is pivotal for next‐generation cobalt‐free batteries intended for fast‐charging EV platforms and grid applications. These convergent findings—from FTIR and TGA characterizations, through swelling, FESEM‐derived porosity/tortuosity metrics, and ICI analyses, to galvanostatic cycling—identify the TRD202A + carbon‐coated foil combination as the best‐performing configuration among those investigated for achieving durable, high‐energy LNMO cathodes adopting a sustainable, waterborne electrode manufacturing process. It is worth noting that the present study was deliberately designed as a half‐cell investigation in order to isolate the effect of binder chemistry and current‐collector engineering on LNMO cathode behavior without the confounding degradation pathways introduced by a graphite counter‐electrode (e.g., crosstalk between cathode‐derived transition‐metal dissolution and anode SEI degradation). Likewise, no PVdF/NMP‐based benchmark was processed in parallel since the explicit aim of this work is to advance water‐processable alternatives to the PVdF/NMP system rather than to reproduce it; it is worth noting that the initial capacities achieved here with the TRD202A/CCAF combination (~130−135 mAh g^−1^ at C/10–C/5) are already comparable to those reported for well‐optimized PVdF‐based LNMO half‐cells reported in the literature. Full‐cell validation against graphite anodes, together with a systematic evaluation of PVdF‐based controls processed under identical conditions, represents the natural next step and is the subject of ongoing work. Interfacial chemical characterization (e.g., XPS) of the binder‐collector interface and systematic optimization of the binder‐ratio composition (e.g., via mixture design methodologies) are likewise identified as priority directions for future work.

## Funding

This study was supported by HORIZON EUROPE Framework Programme (101069707).

## Supporting information

Supplementary Material

## Data Availability

The data that support the findings of this study are available from the corresponding author upon reasonable request.
