# Supplementary material for: Unlocking High‐Voltage Aqueous LiNi0.5Mn1.5O4 Cathodes Through Binder‐Collector Interfacial Design
Source: Small Sci. 2026 Sep 25;6(9):e70406. doi: 10.1002/smsc.70406 (PMC13618630; doi:10.1002/smsc.70406)
Supplement: Supplementary file 1 [file SMSC-6-e70406-s001.pdf]

Unlocking High-Voltage Aqueous $LiNi_{0.5}Mn_{1.5}O_4$ Cathodes through Binder-Collector Interfacial Design

Giorgio Montinaro*, Daniele Versaci, Piera Di Prima, Julia Amici, Federico Bella*, Silvia Bodoardo

Department of Applied Science and Technology, Politecnico di Torino, Corso Duca degli Abruzzi, 24, 10129 Turin, Italy

*Corresponding authors: giorgio.montinaro@polito.it; federico.bella@polito.it

Supporting information

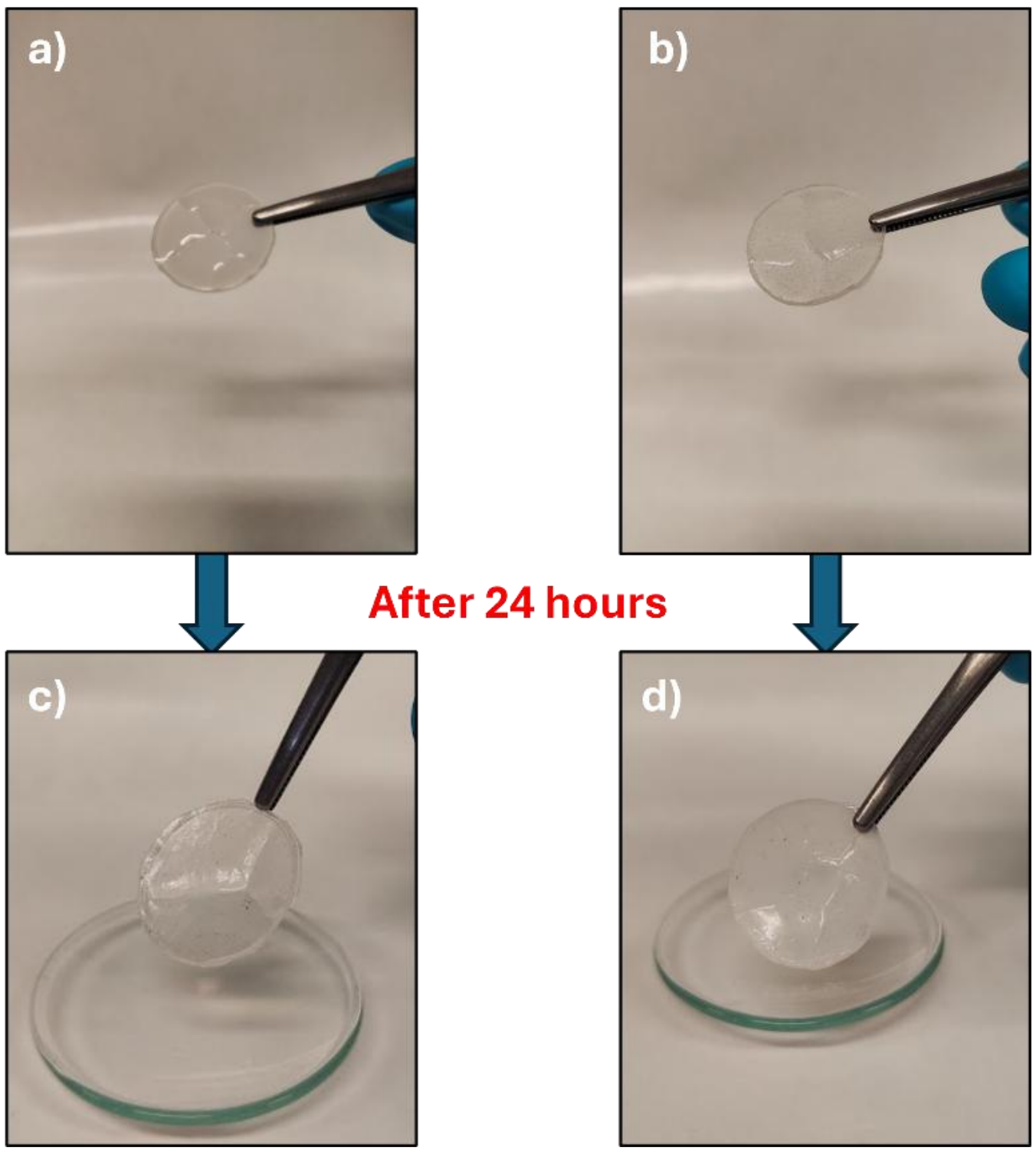

Figure S1 - Photographic comparison of self-standing binder disks (a, b) before and (c, d) after 24 h immersion in LP40 electrolyte: (a, c) SX8684(A)-64 and (b, d) TRD202A. Both formulations retain their original appearance, exhibiting only a slight increase in opacity and no visible change in overall dimensions.

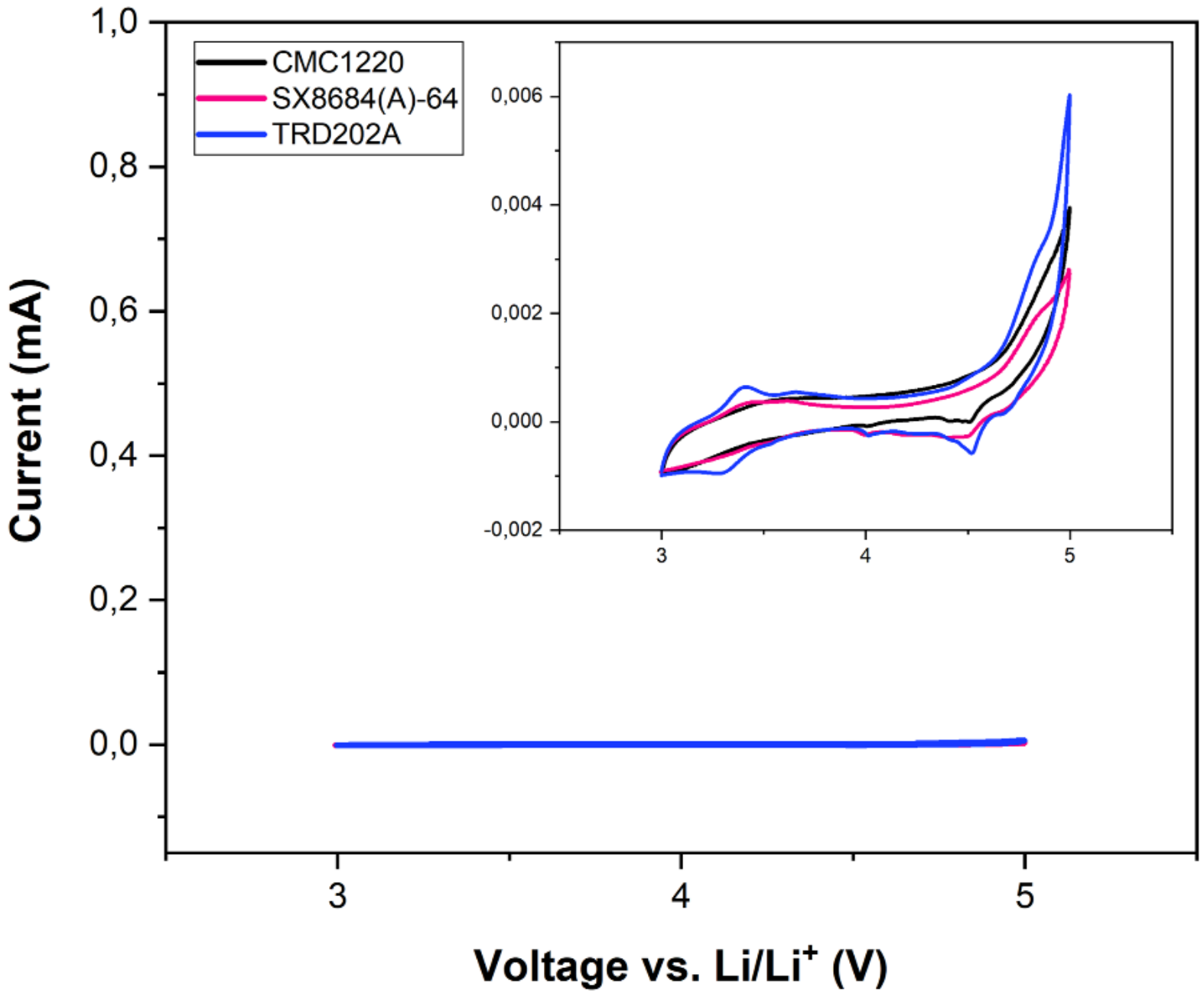

Figure S2 - CV curves for both binder samples (TRD202A and SX8684(A)-64, against CMC1220 reference) performed at a scan rate of 0.1 mV s^{-1}

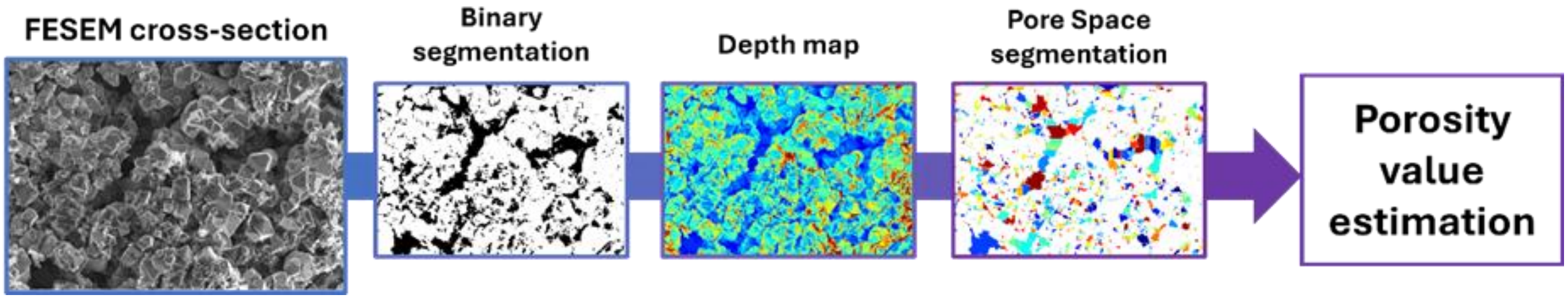

Figure S3 – Flowchart illustrating the image-processing workflow used to quantify porosity from FESEM cross-section micrographs. By applying tailored colour-mapping and segmentation algorithms, solid material and pore spaces are accurately differentiated, enabling precise calculation of the sample porosity.

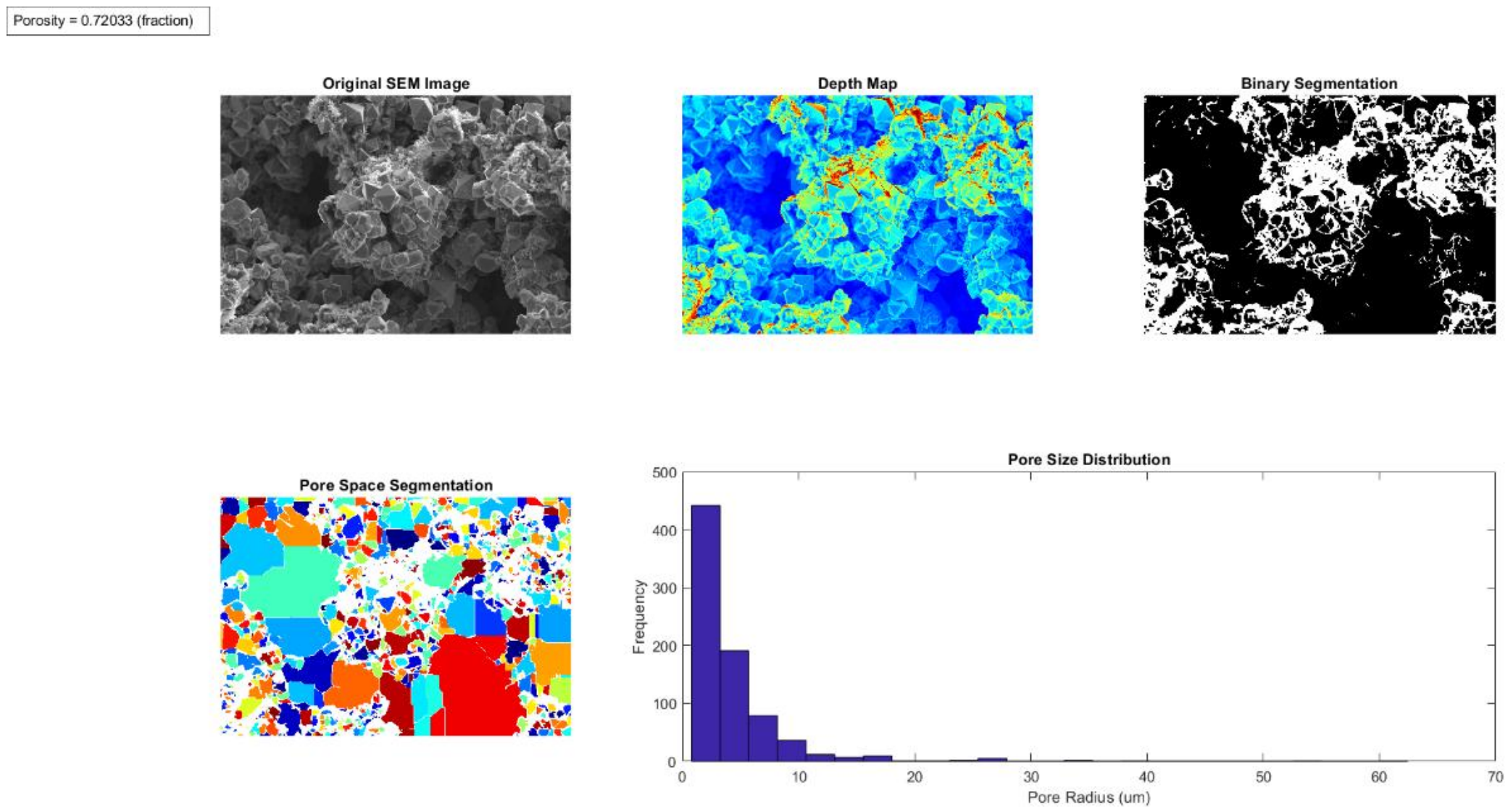

Figure S4 – Example output from the porosity analysis tool, displaying the calculated porosity fraction and pore-size distribution histogram derived from a single FESEM cross-section image

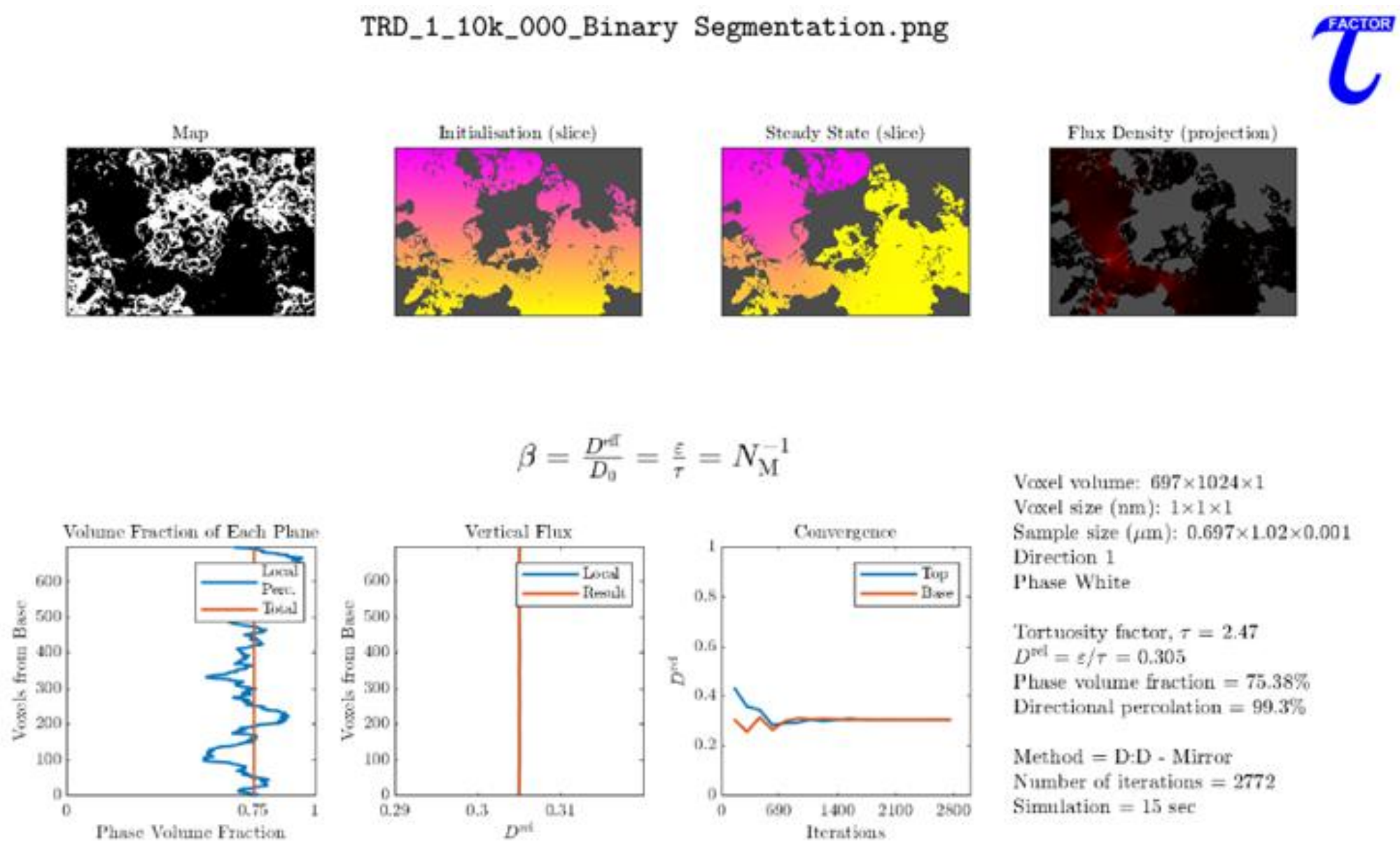

Figure S5– Example output from the MATLAB "τ-factor" script, which computes the electrode tortuosity from a binary-segmented FESEM cross-section image.
